# Non‐Cirrhotic Steatotic Liver Disease is Associated With Impaired Muscle Function: A Cross‐Sectional Study

**DOI:** 10.1002/rco2.70012

**Published:** 2025-10-07

**Authors:** Guillaume Henin, Alexis Goffaux, Salomé Declerck, Stéphanie André‐Dumont, Etienne Pendeville, Maxime Valet, Thierry Lejeune, Géraldine Dahlqvist, Audrey Loumaye, Bernd Schnabl, Peter Stärkel, Nicolas Lanthier

**Affiliations:** ^1^ Laboratoire de gastro‐entérologie (GAEN) Institut de recherche expérimentale et clinique (IREC), UCLouvain Brussels Belgium; ^2^ Service d'hépato‐gastroentérologie Cliniques universitaires Saint‐Luc, UCLouvain Brussels Belgium; ^3^ Service de kinésithérapie et ergothérapie Cliniques universitaires Saint‐Luc, UCLouvain Brussels Belgium; ^4^ Laboratoire de pathologie neuro‐musculo‐squelettique (NMSK) Institut de recherche expérimentale et clinique (IREC), UCLouvain Brussels Belgium; ^5^ Service de médecine physique et réadaptation Grand Hôpital de Charleroi Charleroi Belgium; ^6^ Service de médecine physique et réadaptation Cliniques universitaires Saint‐Luc, UCLouvain Brussels Belgium; ^7^ Service d'endocrinologie et diabétologie Cliniques universitaires Saint‐Luc, UCLouvain Brussels Belgium; ^8^ Department of Medicine University of California San Diego La Jolla CA USA; ^9^ Department of Medicine VA San Diego Healthcare System San Diego CA USA

**Keywords:** alcohol‐related liver disease, isokinetic dynamometer, liver frailty index, liver–muscle axis, metabolic dysfunction‐associated steatotic liver disease, muscle function, steatotic liver disease

## Abstract

**Background:**

Impaired muscle function is frequent in cirrhosis and potentially participates in liver disease progression. Data from non‐cirrhotic patients with steatotic liver disease (SLD) are lacking. Our aims were to determine if muscle function was impaired in a non‐cirrhotic cohort of patients with SLD and if the SLD subtype and severity were associated with impaired muscle function.

**Methods:**

Patients with SLD and controls were prospectively recruited. Liver disease was assessed by imaging, vibration‐controlled transient elastography and non‐invasive tests. Muscle function was assessed by the liver frailty index (LFI) and isokinetic dynamometer. Diet and physical activity habits were recorded using dedicated questionnaires to measure the energetic balance.

**Results:**

One‐hundred and fifty patients with non‐cirrhotic SLD (75 patients with metabolic dysfunction‐associated steatotic liver disease [MASLD] and 75 patients with alcohol‐related liver disease [ALD]) and 30 non‐SLD controls were prospectively recruited. The LFI negatively correlated to lower limb muscle strength assessed by isokinetic dynamometer in all participant groups (*r* = 0.82 in the control group; *r* = 0.69 in the pooled SLD group—*p* < 0.0001). Both SLD groups showed muscle strength impairment assessed by the LFI compared to age‐ and gender‐matched controls. In multivariate analysis, the presence of SLD was associated with impaired muscle function independently of age, BMI and energetic balance, with a higher risk related to ALD.

**Conclusions:**

Patients with SLD already show impaired muscle function compared to controls independently of age, gender and energetic balance. Taken together, our data support a potential disruption of the liver–muscle axis already occurring in SLD prior to cirrhosis.

**Trial Registration:**

ClinicalTrials.gov identifier: NCT06514300

**Abbreviations**
ALD: alcohol‐related liver disease
CAP: controlled‐attenuation parameter
HOMA‐IR: homeostasis model assessment for insulin resistance
ID: isokinetic dynamometry
LFI: liver frailty index
LSM: liver stiffness measurement
MASLD: metabolic dysfunction‐associated steatotic liver disease
MetALD: metabolic and alcohol‐related steatotic liver disease
SLD: steatotic liver disease
T2DM: type 2 diabetes mellitus
VCTE: vibration‐controlled transient elastography

## 1 Background

Steatotic liver disease (SLD) is the most prevalent chronic liver disease worldwide. SLD is characterized by an excessive liver lipid content, or liver steatosis. SLD includes three major clinical entities according to their aetiology: metabolic dysfunction–associated steatotic liver disease (MASLD), alcohol‐related liver disease (ALD) and a mixed entity characterized by the co‐existence of both conditions (MetALD) [1]. Besides liver‐related complications, MASLD associates with extra‐hepatic complications including muscle function impairment related to liver disease severity [2]. Enhancing muscle function through exercise promotes liver disease remission in MASLD [3] whereas in ALD, muscle changes have been poorly investigated [4]. These observations lead to the hypothesis of a bidirectional crosstalk between liver and skeletal muscles, but mechanisms involved remain poorly known [5]. Impaired muscle function in chronic liver diseases was firstly reported in cirrhosis whatever the cause [6, 7, 8]. It is highly prevalent in cirrhosis with worsened post‐liver transplant outcomes [9]. Scores including the liver frailty index (LFI) were developed to screen patients with cirrhosis for homeostasis disruption resulting in increased vulnerability, or frailty on the liver transplant waiting list [10]. This frailty is highly related to muscle function impairment only roughly assessed by the LFI. Using the LFI consisting of simple muscle function tests, the prevalence of frailty in cirrhosis ranks from 10% to 58.8% [11]. Other techniques exist to more accurately assess muscle function including isokinetic dynamometry (ID). ID dynamically measures muscle strength at a constant velocity according to the angular position [12]. Knee extension strength assessed by ID predicts frailty in the elderly and is a better prognosis marker in digestive oncology than grip strength alone [13, 14].

Muscle function data in non‐cirrhotic patients with SLD are scarce and almost no data are available in ALD [4]. In non‐cirrhotic stages of MASLD, frailty is reported as potentially present but mainly in the elderly [15, 16]. Several mechanisms, including a high‐calorie diet with reduced physical activity [17] and insulin resistance [18] could explain why impaired muscle function is not just a consequence of cirrhosis but could occur earlier because of these deleterious factors. In ALD, the myotoxicity of alcohol may also be a causal mechanism [19]. Our hypothesis is that patients with SLD might exhibit a liver–muscle axis disruption at non‐cirrhotic stage, resulting in impaired muscle function compared to non‐SLD subjects. We also hypothesise that this muscle function impairment differs according to SLD subtype due to metabolic differences and alcohol‐related myotoxicity. The LFI might be an alternative to ID, the gold standard technique, for muscle function assessment in this population that we could easily implement in our clinical practice.

Our aims were to assess (i) the relevance of the LFI as a muscle function assessment tool in a non‐cirrhotic SLD cohort by investigating its correlation with ID data and (ii) the association between impaired muscle function and the presence of SLD and its severity.

## 2 Patients and Methods

### 2.1 Recruitment and Definitions

We conducted a prospective observational monocentric study at the Cliniques Universitaires Saint‐Luc, Brussels (Belgium) from April 2022 to December 2024. Adult patients (18–70 years old) with MASLD were recruited from outpatient clinics while patients with ALD were recruited from the alcohol withdrawal unit. Liver steatosis was diagnosed by abdominal imaging and confirmed by controlled attenuation parameter (CAP) (see Liver Disease Assessment section). The SLD subtype and cardiometabolic criteria were defined according to the consensus on SLD (Figure S1) [1]. Patients with steatosis and at least one cardiometabolic criterion were classified as MASLD. Patients with steatosis and an alcohol consumption above > 20 g/day for women and > 30 g/day for men were classified as ALD. In the case of alcohol consumption and cardio‐metabolic criteria, patients were classified as MetALD if consumption was between 20 and 50 g/day in women, and between 30 and 60 g/day in men. Above this level of consumption, patients were classified as ALD even in the presence of cardio‐metabolic criteria [1]. Type 2 diabetes (T2DM) was defined as taking hypoglycaemic drugs [20]. Insulin resistance was assessed in non‐diabetic patients by the homeostasis model assessment for insulin resistance (HOMA‐IR). Exclusion criteria included comorbidities other than T2DM affecting muscle function (e.g., osteoarticular and neurological disorders, malignancies). Control subjects defined by the absence of SLD according to the SLD consensus were recruited in the same centre to assess the impact of the SLD itself on muscle function (Figure S1) [21]. Sample size calculation was not performed due to the lack of published data on the topic. Detailed information about the course of the study was given and written informed consent was obtained. The study was approved by the ethical committee of the Cliniques universitaires Saint‐Luc (2014/15OCT/514 & 2014/14AOU/438) in accordance with the declarations of Helsinki and Istanbul. The results of this study are reported according to the STROBE checklist (see supplementary). This trial is registered in ClinicalTrials.gov (NCT06514300).

### 2.2 Liver Disease Assessment

**FIGURE 1:**
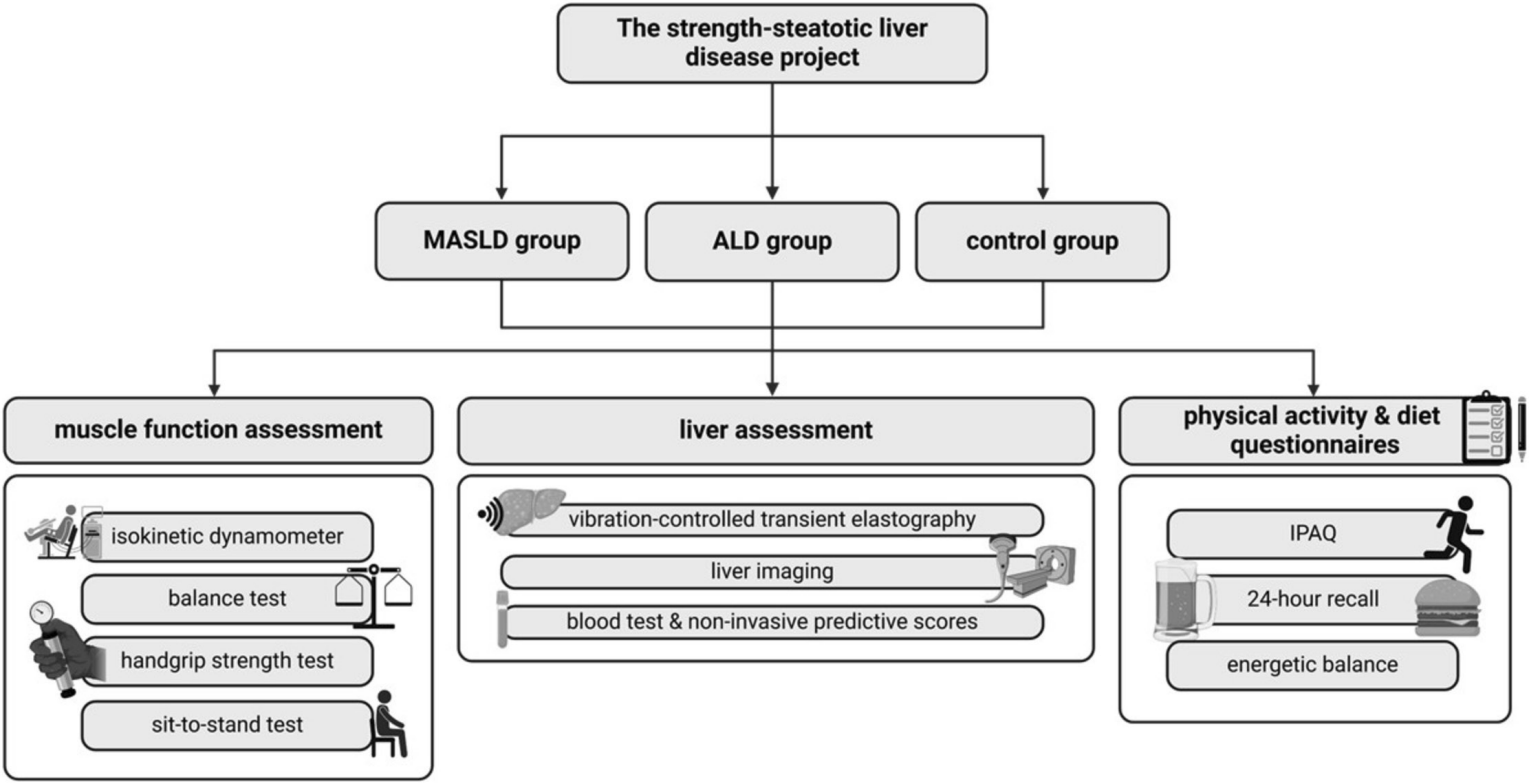
Study design. ALD, alcohol‐related liver disease; IPAQ, international physical activity questionnaire; MASLD, metabolic dysfunction‐associated steatotic liver disease.

SLD severity was assessed by non‐invasive scores and vibration‐controlled transient elastography (VCTE) (Figure 1). Non‐invasive scores for liver steatosis and liver fibrosis were calculated by combining biological and anthropometric data collected from medical files. All patients underwent VCTE (Fibroscan) to measure the CAP (positively correlated to liver steatosis) and liver stiffness measurement (LSM) (positively correlated to fibrosis) in a fasted condition [21]. LSM and CAP data were also converted to fibrosis and steatosis grades using cut‐offs specific to each SLD subtype [22, 23] (Table S1). Patients with LSM values suggestive of cirrhosis (classified F4, Table S1) or signs of cirrhosis or portal hypertension at imaging were excluded. LSM and bio‐anthropometric data were combined to measure the Agile3+ score [24].

### 2.3 Muscle Function Assessment

Muscle function was assessed using the LFI and ID (Figure 1). The gender‐normalised LFI was calculated using three muscle function tests as previously described [10]: dominant handgrip strength, sit‐to‐stand and balance tests (see supplementary). Mean dominant grip strength (Jamar dynamometer) was measured after three consecutive grips [10]. For the sit‐to‐stand test, timed patients had to complete five sit‐to‐stands in a row as quickly as possible. The balance test measures in seconds if the patient can keep balance for 10 s in three positions: feet side by side, semi‐tandem and tandem. The results from the dominant hand grip strength, sit‐to‐stand and balance tests were combined to calculate the gender‐normalised LFI using the following formula: *(−0.330 × gender—mean grip strength) + (−2.529 × number of chair stands per second) + (−0.040 × balance time) + 6* [10]. Importantly, the LFI was used as a continuous variable to assess global muscle function and not as a categorical variable defining frailty.

ID (Cybex) was used to measure mean peak torques of thigh muscles during concentric knee extension and flexion, and leg muscles during concentric ankle plantar and dorsal flexion after a 10‐min warm‐up of cycle ergometer. Speed limit was gradually decreased to 60° per second during knee extension/flexion and 30° per second during plantar/dorsal ankle flexions. Patients executed five recorded repetitions of each movement preceded by four unrecorded adaptation rehearsals at the same speed. Higher peak torques of each repetition at the lowest speed were extracted to calculate the mean peak torque expressed in Newton‐metre reflecting muscle function.

Diet habits and physical activity level were assessed using dedicated questionnaires, notably to measure the energetic balance (see supplementary) [25]. All participants underwent the examinations described above on the same day to avoid bias secondary to potential changes in body composition.

## 3 Statistics

Continuous variables are presented as medians with range values (minimum/maximum) and categorical variables as percentages. The distribution of continuous variables was tested for normality and lognormality using the Shapiro–Wilk test. Outliers were identified and excluded using the ROUT method. Correlation analyses were performed using the Pearson or Spearman tests. Student's *t*‐test or Mann–Whitney *U* tests were used for two‐group comparisons and one‐way ANOVA or Kruskal–Wallis tests for more than two groups according to variable distribution types. *p*‐Value was adjusted for multiple comparisons using Tukey's or Dunn's method. Chi‐square test was used for categorical variable comparisons. Multiple logistic regression was performed using the intercept model after multicollinearity analysis. Statistical tests and graphs were generated on GraphPad Prism version 10.2.3 (GraphPad Software, Boston, Massachusetts USA).

## 4 Results

### 4.1 Study Population

#### 4.1.1 Demographics and Biochemistry

**FIGURE 2:**
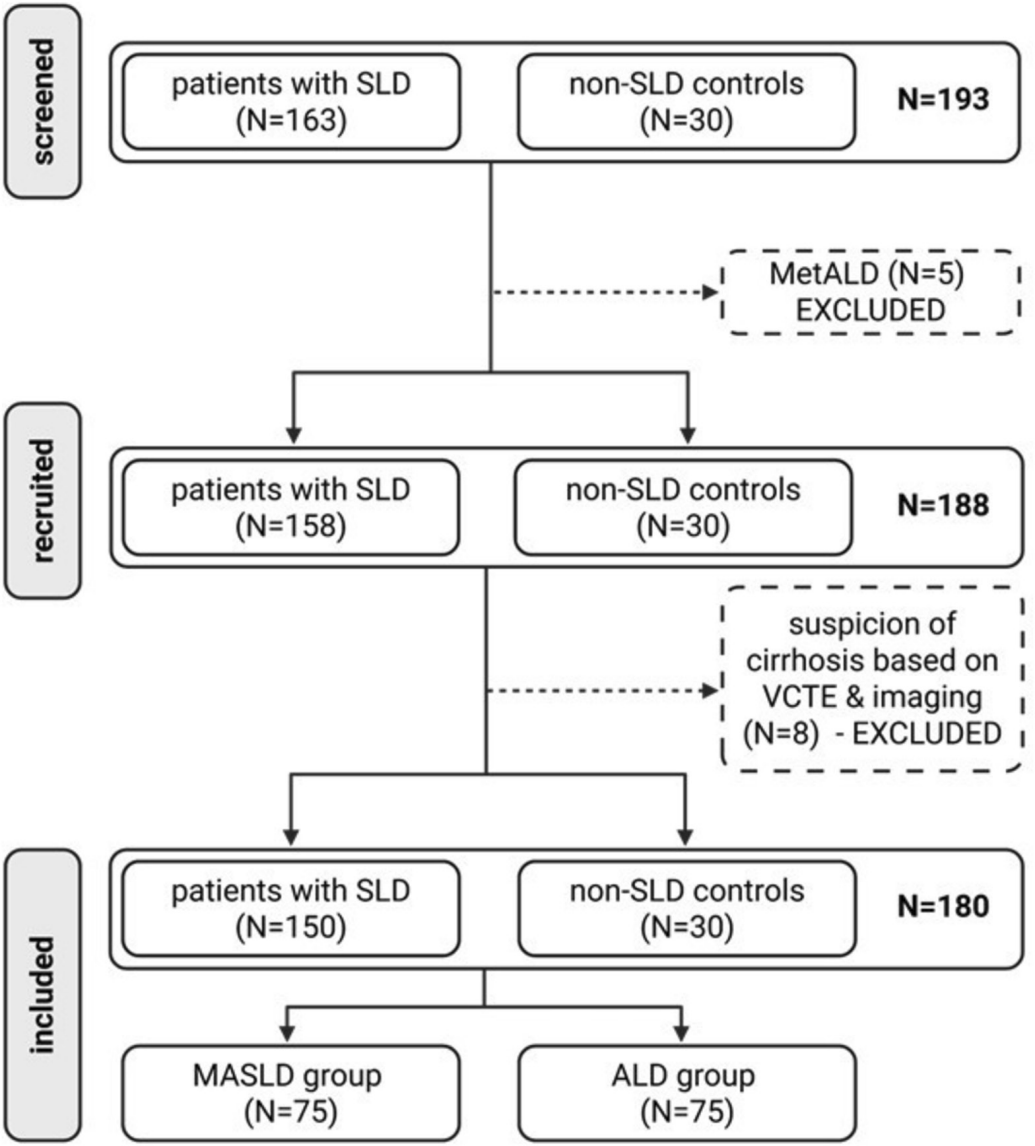
Study flow‐chart. ALD, alcohol‐related liver disease; MASLD, metabolic dysfunction‐associated steatotic liver disease; MetALD: metabolic and alcohol‐related liver disease; SLD, steatotic liver disease; VCTE, vibration‐controlled transient elastography.

One hundred and sixty‐three patients with SLD were prospectively recruited, including 81 patients with MASLD, 77 patients with ALD and five patients classified as MetALD (Figure 2). Patients with MetALD were excluded considering the small sample size and to study the specific effect of metabolic disease and alcohol separately. Six patients were further excluded due to high LSM compatible with cirrhosis (Figure 2). The final study cohort consisted of 150 patients with 75 patients in the MASLD group and 75 in the ALD group (Figure 2). Liver steatosis was diagnosed by ultrasound in 93% (140/150), computed tomography in 5% (8/150) and magnetic resonance imaging in 2% (2/150) of cases. All groups were age‐balanced (*p* = 0.037 but without any difference between groups) whereas mean body mass index was higher in the MASLD group (Table 1, Figure S2A,C). T2DM was more prevalent in the MASLD group (Table 1). The ALD group showed a higher proportion of male gender (Figure S2B) and typical stigmas of chronic alcohol consumption: higher aspartate to alanine amino‐transferase ratio, serum ferritin and gamma glutamyl transferase levels compared to the MASLD group. Mean serum levels of total and HDL‐cholesterol were also higher in the ALD group (Figure S2F). Albumin serum level was similar between groups (Table S2).

**TABLE 1:** Demographics and clinical data.

Variables	Control group (*N* = 30)		MASLD group (*N* = 75)		ALD group (*N* = 75)		*p*‐Value
	Median	[min; max]	Median	[min; max]	Median	[min; max]	
**Anthropometric & clinical data**							
Age (years)	53.5	[25; 70]	57	[27; 70]	51	[27; 70]	0.037
Male gender – *n* (%)	12 (40)	—	39 (52)	—	54 (72)	—	0.004^β, γ^
Weight (kg)	65	[47.3; 85.6]	94.5	[62.3; 160]	78.5	[10.3; 120]	< 0.001^α, β, γ^
Body mass index (kg/m^2^)	22.2	[19; 26]	32.1	[22; 56]	25.7	[17; 40]	< 0.001^α, β, γ^
Type 2 diabetes – *n* (%)	0	—	31 (41)	—	2 (3)	—	< 0.001^β, γ^
Waist circumference (cm)	79	[62; 95]	109	[83; 153]	99	[66; 127]	< 0.001^α, β, γ^
Employment rate – *n* (%)	21 (70)	—	42 (56)	—	34 (45)	—	0.022^β^
**Cardiometabolic risk factors**							
0 – *n* (%)	23 (77)	—	0	—	8 (11)	—	< 0.001^α, β^
1 – *n* (%)	6 (20)	—	6 (8)	—	17 (23)	—	0.011^γ^
2 – *n* (%)	1 (3)	—	12 (16)	—	20 (27)	—	< 0.001^α, β^
3 – *n* (%)	0	—	22 (29)	—	16 (21)	—	< 0.001^α, β^
4 – *n* (%)	0	—	14 (19)	—	10 (13)	—	< 0.001^α, β^
5 – *n* (%)	0	—	21 (28)	—	4 (5)	—	< 0.001^α, β, γ^

*Note:* Significant comparisons between controls and MASLD (^α^), controls and ALD (^β^), MASLD and ALD (^γ^); ALD, alcohol‐related liver disease; MASLD, metabolic dysfunction‐associated steatotic liver disease.

#### 4.1.2 Liver Disease Severity Assessment

Mean Fibrosis‐4 index was higher in the ALD group. No difference was observed for mean non‐alcoholic fatty liver disease fibrosis, Hepamet fibrosis, Agile3+ scores and LSM (Table S2, Figure S3A). A higher proportion of patients with ALD were classified as F0–F1 based on LSM (Figure S3B). Mean CAP was higher in the MASLD group compared to the ALD group (Figure S3C) but with equal proportions of steatosis grades (Figure S3D).

#### 4.1.3 Physical Activity and Dietary Data

Physical activity assessed by the international physical activity questionnaire showed no difference in calorie expenditure related to exercise between study groups (Table 2). The control group exercised more during leisure time than the SLD groups (Figure S4). For eating habits assessed by the 24‐h recall questionnaire, patients with ALD consumed more calories despite a mean BMI lower than patients with MASLD. This difference in calorie intake was mainly explained by a higher consumption of alcoholic beverages (952.4 ± 651.2 in ALD vs. 33.9 ± 81.5 kcal/day in MASLD groups, *p* < 0.0001). MASLD and control groups showed no difference in daily calorie intake. Patients with ALD also consumed lower proportions of proteins, lipids, carbohydrates and fibres than other groups (Table 2, Figure S5).

**TABLE 2:** Muscle function, diet and physical activity data.

Variables	Control group (*N* = 30)		MASLD group (*N* = 75)		ALD group (*N* = 75)		*p*‐Value
	Median	[min; max]	Median	[min; max]	Median	[min; max]	
**Muscle function data**							
Dominant handgrip (kg)	29.3	[15; 66]	28	[8; 62]	34	[12; 65]	0.245
Sit‐to‐stand (s)	5.4	[3.4; 11]	8.7	[3.9; 22.3]	8.9	[3.7; 23.6]	< 0.001^α, β^
Balance test (s)	30	[30; 30]	30	[24; 30]	30	[20; 30]	0.311
Liver frailty index	2.3	[0.4; 3.6]	3.1	[1.7; 4.6]	3.2	[1.1; 4.7]	< 0.001^α, β^
Knee extension (Nm) (*)	92.4	[45.9; 192.7]	92.8	[23.4; 262.4]	102.2	[22.5; 209.7]	0.646
Knee flexion (Nm) (*)	61.9	[33.1; 115.5]	55.4	[23.3; 151.9]	61.9	[8.2; 115.9]	0.198
Ankle plantar flexion (Nm) (*)	51.7	[18.3; 123.8]	35.9	[8.4; 134.4]	42.5	[8.9; 94.4]	0.015^α^
Ankle dorsal flexion (Nm) (*)	15.3	[5.8; 33]	15.7	[3.3; 38.5]	15.6	[3; 29.7]	0.416
**24‐h recall questionnaire**							
Total calorie intake (kcal)	2042	[705; 3671]	1715	[319; 3377]	2314	[608; 6666]	< 0.001^β, γ^
Proteins (% TCI)	16	[10; 34]	16	[9; 40]	11	[4; 29]	< 0.001^β, γ^
Carbohydrates (% TCI)	36.5	[14; 61]	42	[12; 64]	29	[5; 59]	< 0.001^β, γ^
Added sugars (% TCI)	11.5	[0; 28]	6	[0;61]	8.5	[0; 40]	< 0.001^α, γ^
Lipids (% TCI)	41	[15; 88]	37	[16; 62]	23	[2; 51]	< 0.001^β, γ^
Saturated fatty acids (% TCI)	14.5	[2; 29]	16	[3; 36]	8	[1; 31]	< 0.001^β, γ^
Fibres (% TCI)	2.4	[0.7;9]	1.8	[0.4; 4.7]	0.8	[0.1; 3.8]	< 0.001^β, γ^
Alcohol (% TCI)	0	[0; 15]	0	[0; 15]	35	[0; 85]	< 0.001^β, γ^
**International physical activity questionnaire**							
Total MET (MET‐min/week)	3107	[429; 11 001]	1908	[49.5; 10 533]	1556	[0; 10 548]	0.073
Work (% total MET) (**)	0	[0; 19.1]	0.5	[0; 100]	13.8	[0; 100]	0.124
Transport (% total MET)	10.4	[0; 78.4]	6	[0; 100]	8.5	[0; 31.4]	0.303
Domestic (% total MET)	21.1	[0; 55.6]	44.4	[0; 100]	23.3	[0; 100]	0.005^α^
Leisure (% total MET)	50.7	[0; 100]	8.3	[0; 100]	16.8	[0; 100]	< 0.001^α, β^

*Note:* Significant comparisons between controls and MASLD (^α^), controls and ALD (^β^), MASLD and ALD (^γ^); ALD, alcohol‐related liver disease; MASLD, metabolic dysfunction‐associated steatotic liver disease; MET, metabolic equivalent; TCI, total calorie intake; (*) right‐side isokinetic dynamometer data; (**) data from employed participants.

### 4.2 LFI is a Fast and Easy Tool Well Correlated to Isokinetic Dynamometer

In all study groups, the LFI negatively correlated to ID data from all lower limb muscle groups assessed (Table 3). Knee extension strength correlated best with grip strength in the control group (*r* = 0.82, *p* < 0.0001), the MASLD group (*r* = 0.76, *p* < 0.0001), the ALD group (*r* = 0.63, *p* < 0.0001) and the pooled SLD group (*r* = 0.69, *p* < 0.0001). These correlation analyses were performed with ID data from the right lower limb, considering higher mean peak torques from right quadriceps in the MASLD group alone (Figure S6).

**TABLE 3:** Muscle function tests correlation matrix.

	Control group (*N* = 30)			MASLD group (*N* = 75)			ALD group (*N* = 75)			MASLD & ALD groups (*N* = 150)		
	LFI	Handgrip	STS	LFI	Handgrip	STS	LFI	Handgrip	STS	LFI	Handgrip	STS
KE^†^	−0.53**	0.82****	−0.37*	−0.54****	0.76****	−0.36**	−0.52****	0.63****	−0.44****	−0.50****	0.69****	−0.38****
KF^†^	−0.56**	0.86****	−0.40*	−0.54****	0.72****	−0.36**	−0.58****	0.61****	−0.50****	−0.58****	0.66****	−0.45****
APF^#^	−0.51**	0.81****	−0.42*	−0.58****	0.68****	−0.40***	−0.47****	0.48****	−0.47***	−0.56****	0.58****	−0.46****
ADF^#^	−0.40*	0.75****	−0.31	−0.28*	0.66****	−0.15	−0.30**	0.43****	−0.26*	−0.31***	0.54****	−0.18*

Abbreviations: ADF, ankle dorsal flexion strength; ALD, alcohol‐related liver disease; APF, ankle plantar flexion strength; KE, knee extension strength; KF, knee flexion strength; LFI, liver frailty index; MASLD, metabolic dysfunction‐associated steatotic liver disease; STS, sit‐to‐stand test.

* *p*‐Value < 0.05.

** *p*‐Value < 0.01.

*** *p*‐Value < 0.001.

**** *p*‐Value < 0.0001.

^†^ Pearson's correlations.

^#^ Spearman's correlations.

### 4.3 Patients With MASLD or ALD Have the Same Muscle Function

**FIGURE 3:**
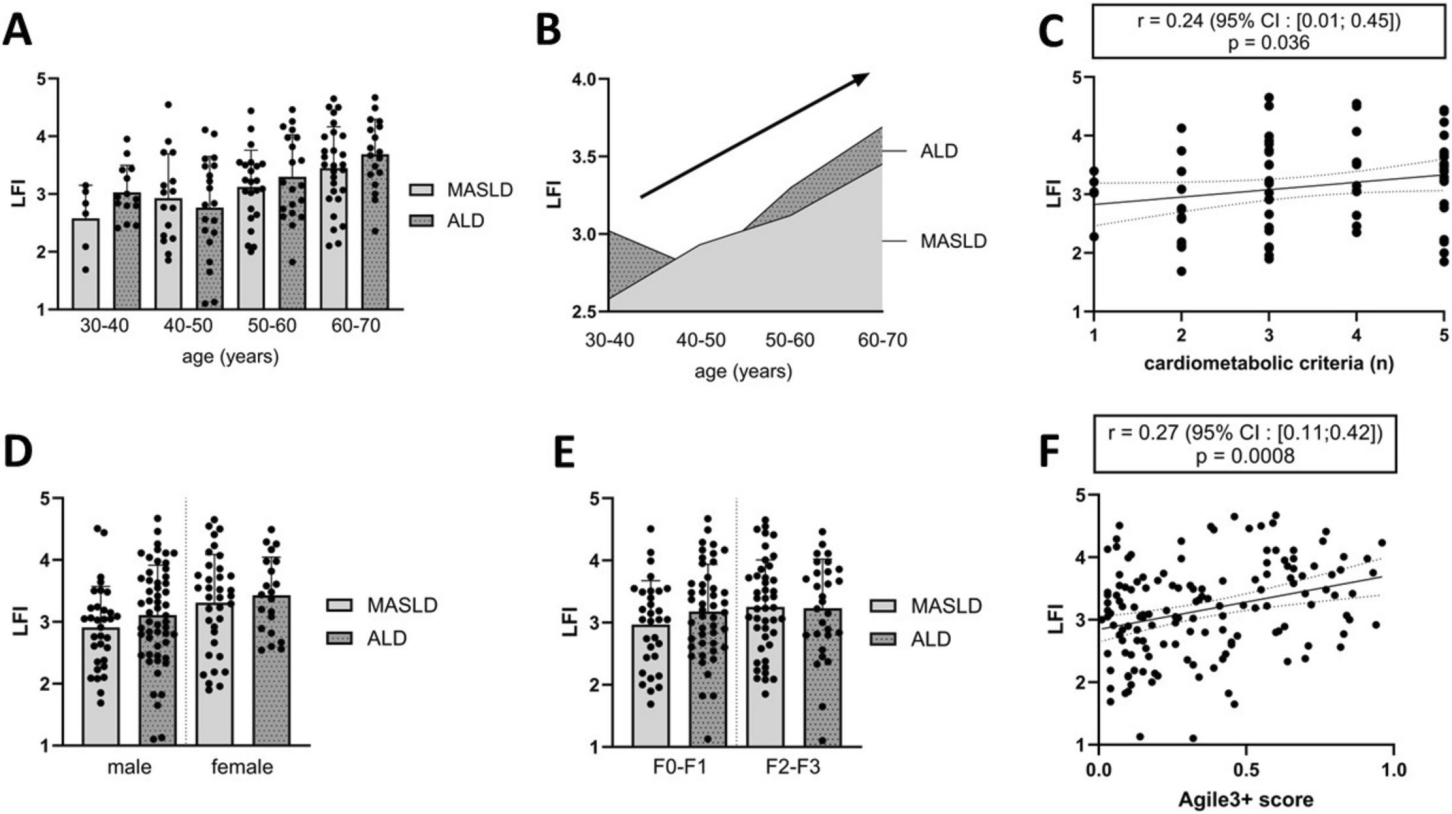
Muscle function comparison between MASLD and ALD groups. Mean LFI between 10‐year age matched groups (A); relation of LFI and age in MASLD and ALD groups (B); correlation of LFI and cardiometabolic criteria in MASLD (C); mean LFI between age and gender‐matched groups (D); mean LFI between groups matched for fibrosis grades assessed by transient elastography (E); correlation of LFI and Agile3+ score in pooled patients with MASLD and ALD (F). ALD, alcohol‐related liver disease; LFI, liver frailty index; MASLD, metabolic dysfunction‐associated steatotic liver disease. (A, D, E) Student's *t* tests; (C, F), Spearman's correlations.

Comparing SLD groups, we report no difference in terms of LFI despite drastically different metabolic profiles (Table 2). No differences for dominant handgrip strength, sit‐to‐stand and balance tests were observed between groups. There was no difference in ID data for all lower limb muscles strength assessed by ID between groups (Table 2). This result was confirmed after matching by 10‐year age subgroups (Figure 3A) only highlighting age‐related physiological muscle function impairment (Figure 3B). The same results were obtained after adjustment for age and gender (Figure 3D). Interestingly, the LFI positively correlated with the number of cardiometabolic criteria present in patients with MASLD alone (Figure 3C), suggesting a cumulative negative effect of cardiometabolic impairment on global muscle function. In terms of liver disease severity, we observed no difference in mean LFI according to fibrosis grades assessed by LSM (Figure 3E). However, the Agile 3+ score combining LSM and bio‐anthropometric data was positively correlated with the LFI in pooled patients with SLD (Figure 3F).

### 4.4 Patients With SLD Face Impaired Muscle Function Compared to Controls

**FIGURE 4:**
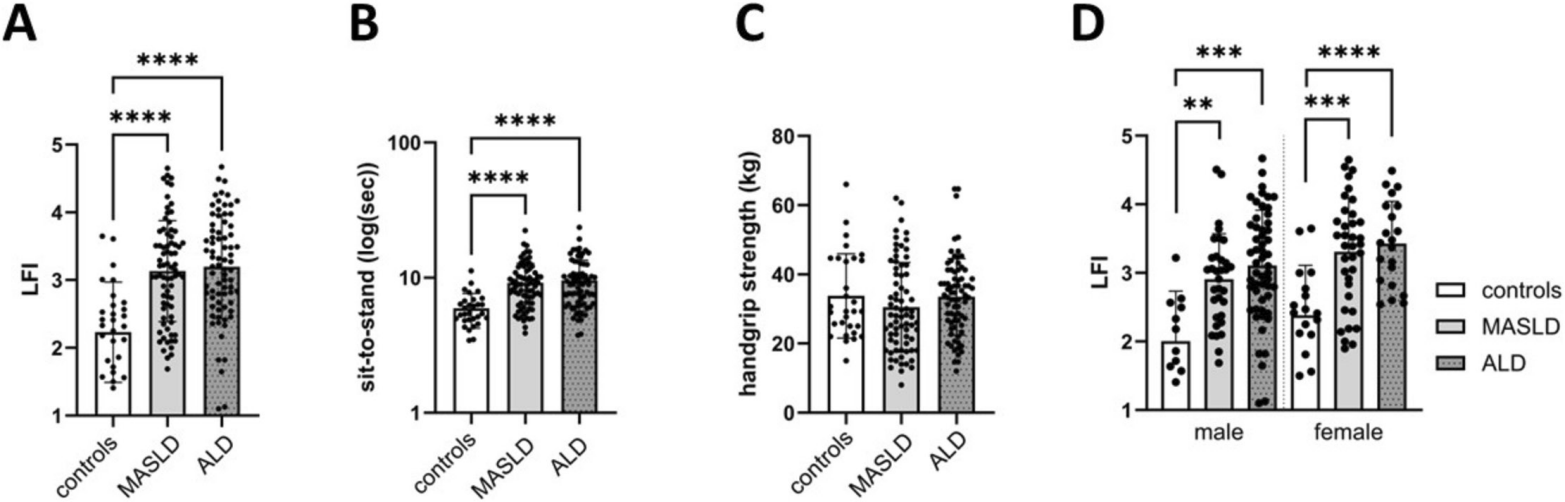
Muscle function comparison between control, MASLD and ALD groups. Mean LFI (A), sit‐to‐stand test (B) and handgrip strength (C) between unmatched groups; (D) mean LFI between age and gender‐matched groups. ALD, alcohol‐related liver disease; F, fibrosis grade; LFI, liver frailty index; MASLD, metabolic dysfunction‐associated steatotic liver disease. (A–D) One‐way ANOVA. **p‐value < 0.01; ***p‐value < 0.001; ****p‐value < 0.0001.

Patients with MASLD and ALD had a higher LFI compared to controls, highlighting an impaired global muscle function already in non‐cirrhotic patients (Table 2, Figure 4A). This result was confirmed after matching SLD patients and controls for age and gender (Figure 4D). The sit‐to‐stand is the only variable explaining this difference (Table 2, Figure 4B). However, we did not observe any difference in terms of lower limb muscle function assessed by ID and grip strength (Table 2). Taken together, these data suggest that the muscle function assessed by the LFI is impaired in non‐cirrhotic patients with SLD compared to controls, while lower limb muscle function and grip strength are preserved.

### 4.5 The Presence of SLD is Strongly Associated With Global Muscle Function Impairment Assessed by the LFI

Multivariate logistic regression was used to investigate the independent relationship between SLD and decreased global muscle function. Decreased muscle function was defined by a LFI equal to or higher than its 25th percentile (LFI = 2.63) from pooled patients with SLD (*N* = 150) (Figure S7A). Seven controls (7/30—23%) and 111 patients with SLD (111/150—74%) were classified in the decreased muscle function group (LFI ≥ 2.63) to build multivariate logistic regression models. Interestingly, the presence of SLD remained significantly associated with decreased muscle function (OR = 10.3, *p* < 0.001) after adjustment for age, BMI, T2DM, LSM, smoking status and energetic balance (Table 4). A second model was built to assess the odd ratio related to severely decreased global muscle function according to SLD subtypes. Severely decreased muscle function was defined by a LFI equal to or higher than its 75th percentile (LFI ≥ 3.705) from pooled patients with SLD (*N* = 150) (Figure S8A). Thirty‐seven patients had severely decreased muscle function (37/150—25%). Comparing patients with MASLD and ALD, ALD better predicted decreased global muscle function than MASLD (OR = 4.1, *p* = 0.004) after adjustment for the same variables (Table 4). In both models, age was the only other significant regressor explaining muscle function. Both model accuracies were good (AUC = 0.78 for SLD versus non‐SLD groups (Figure S8C); AUC = 0.73 for MASLD versus ALD groups (Figure S8C),*p* < 0.001) with low probability of multicollinearity (Figures S7D, S8D). Gender was not included in these models because the LFI is already gender normalised.

**TABLE 4:** Multiple logistic regression analyses for decreased muscle function according to the presence of steatotic liver disease in the whole cohort and to ALD in the steatotic liver disease cohort.

	Steatotic liver disease group (*N* = 150) versus control group (*N* = 30)					MASLD group (*N* = 75) versus ALD group (*N* = 75)				
	LFI < 2.63		LFI ≥ 2.63		*p*‐Value	LFI < 3.70		LFI ≥ 3.70		*p*‐Value
	OR	95% CI	OR	95% CI		OR	95% CI	OR	95% CI	
Unadjusted	0.1	[0.04; 0.26]	9.3	[3.9; 25.2]	< 0.001	0.70	[0.33; 1.47]	1.4	[0.68; 3.06]	0.340
Model 1	0.08	[0.03; 0.23]	12.4	[4.4; 38.9]	< 0.001	0.34	[0.12; 0.88]	2.97	[1.13; 8.25]	0.030
Model 2	0.08	[0.03; 0.23]	12.0	[4.3; 37.6]	< 0.001	0.24	[0.07; 0.71]	4.2	[1.40; 14.2]	0.010
Model 3	0.10	[0.02; 0.34]	10.2	[2.9; 39.5]	0.004	0.24	[0.06; 0.81]	4.1	[1.23; 15.3]	0.004

*Note:* Unadjusted analysis for the presence of steatotic liver disease alone (A) or the presence of alcohol‐related liver disease (ALD) alone (B). Model 1 included the presence of steatotic liver disease (A) or ALD (B), age and body mass index; model 2 included the variables used in model 1, type 2 diabetes and liver stiffness; model 3 included the variables used in model 2, active smoking and energetic balance.

Abbreviations: ALD, alcohol‐related liver disease; LFI, liver frailty index; MASLD, metabolic dysfunction‐associated steatotic liver disease; OR: odd ratio.

## 5 Discussion

Our work presents new insights in the field of SLD.

Firstly, the LFI assesses quickly and easily global muscle function, but it was previously neither specifically used in non‐cirrhotic patients with SLD nor compared to a gold‐standard assessment tool. Good correlation rates were previously reported between different frailty scores in patients with and without cirrhosis [11, 15]. But these scores, other than the LFI, often include subjective self‐reported parameters. Our study is the first to confront an objective frailty score with a gold‐standard technique for muscle function assessment, which is ID. We show a high correlation between both techniques, demonstrating the validity of the LFI for rapidly assessing global muscle function. Our work is also original, considering the use of the LFI as a continuous variable. Indeed, LFI cut‐offs defining robust, pre‐frail and frail patients, as described by Lai J. et al., are inappropriate considering that they were established from decompensated cirrhotic cohorts as post‐liver transplant prognostic factors [10].

Second, we are the first to report the absence of difference in terms of muscle function between patients with MASLD and ALD in a large non‐cirrhotic SLD cohort. This finding is surprising considering their specific metabolic features [26] and a significantly decreased daily protein intake in the ALD group as previously reported [25]. A worse muscle function might be expected from patients with ALD considering alcohol‐related myotoxicity [19]. Our multivariate analysis supports this hypothesis with a higher association with decreased global muscle function compared to MASLD independently of age and BMI. Of note, all recruited patients with ALD underwent a systematic screening for peripheral polyneuropathy by neurologists and returned negative. We also report an association between SLD severity assessed by the Agile3+ score and the LFI in pooled patients with SLD. These data highlight impaired muscle function as an extra‐hepatic feature associated with both MASLD and ALD, supporting the consensus gathering them under the common entity of SLD [1, 26].

Third, we show that global muscle function assessed by the LFI is already impaired in non‐cirrhotic patients with SLD compared to age‐ and gender‐matched controls. To our knowledge, only one other study investigated muscle function in patients with chronic liver diseases at a non‐cirrhotic stage using the LFI with similar results [27]. In this cohort of 91 American patients, 42% were classified as pre‐frail (LFI > 3) and 5% were frail (LFI > 4.5) [27]. However, this study included chronic liver diseases of different aetiologies with 29% of previously named non‐alcoholic fatty liver disease and 16% of ALD [27]. This finding is reinforced by our multivariate analysis confirming an independent association between SLD and decreased global muscle function. Differences in physical activity level assessed by the IPAQ might contribute to muscle function impairment observed and the pathogenesis of SLD itself [28]. Although calorie expenditure related to physical activity did not significantly differ, MASLD and ALD groups consumed 38.6% and 49.9% fewer calories respectively than the control group. Further studies objectively measuring energy expenditure related to daily physical activity using accelerometers, pedometers or heart‐rate monitors are needed in the SLD field [29]. Cardiometabolic comorbidities might also impact muscle function independently of the underlying SLD, considering that controls enrolled in this study exhibit fewer comorbidities than both SLD groups.

Our study has several limitations. First, SLD severity was assessed non‐invasively using VCTE and non‐invasive scores. Although liver biopsy remains the gold standard for SLD severity assessment, recent studies reported good prognostic values of LSM in ALD using higher cut‐off values considering that alcohol consumption increases LSM [30]. Second, we did not assess muscle phenotype and were not able to highlight a specific muscle change (e.g., sarcopenia or myosteatosis) linked to muscle function impairment. Studies investigating this association between muscle function and phenotype assessed by qualitative (muscle fat content) and quantitative (lean muscle mass) imaging techniques are required [31]. Normalising muscle function data for lean muscle area using cross‐sectional imaging might better highlight a disruption between muscle mass and muscle function in this population. Third, consumption of alcohol was reported based on the patient's anamnesis. We did not investigate alcohol consumption by objective markers to check the absence of heavy alcohol consumption in patients with MASLD. Finally, we did not investigate MetALD and the potential mixed effect of metabolic syndrome and alcohol on muscle function [32]. A study of muscle function in MetALD remains to be carried out.

Although the impact of impaired muscle function on clinical outcomes remains under investigation in non‐cirrhotic patients with SLD, muscle changes are linked to overall mortality in asymptomatic adults [33]. Investigating skeletal muscle function in these patients is important in light of the current lack of pharmacological treatment approved by regulatory agencies for SLD [34]. Indeed, physical activity has the potential to improve both liver disease and muscle function in MASLD and ALD, in addition to diet and long‐term alcohol abstinence [17, 35, 36]. Screening these patients for decreased muscle function using the LFI could encourage physical activity or their inclusion in exercise medicine programmes, which likely has a positive impact on liver disease progression or could even help to curb the SLD pandemic [37]. Longitudinal studies need to be carried out to see whether these abnormalities are reversible with specific treatment of liver disease.

## Author Contributions

**Guillaume Henin:** conceptualization, data curation, formal analysis, investigation, methodology, project administration, validation, visualization, writing – original draft preparation, writing – review and editing. **Alexis Goffaux:** data curation, investigation, writing – review and editing. **Salomé Declerck:** data curation, investigation, writing – review and editing. **Stéphanie André‐Dumont:** data curation, investigation, writing – review and editing. **Etienne Pendeville:** writing – review and editing. **Maxime Valet:** writing – review and editing. **Thierry Lejeune:** writing – review and editing. **Géraldine Dahlqvist:** writing – review and editing. **Audrey Loumaye:** writing – review and editing. **Bernd Schnabl:** conceptualization, funding acquisition, methodology, resources, supervision, writing – review and editing. **Peter Stärkel:** conceptualization, funding acquisition, methodology, resources, supervision, writing – review and editing. **Nicolas Lanthier:** conceptualization, funding acquisition, investigation, methodology, project administration, resources, supervision, validation, writing – original draft, writing – review and editing.

## Ethics Statement

This study was approved by the ethical committee of the Cliniques universitaires Saint‐Luc (2014/15OCT/514 & 2014/14AOU/438) in accordance with the declarations of Helsinki and Istanbul.

## Consent

Each patient included in this study received detailed oral information and signed a dedicated informed consent previously validated by the ethical committee after a systematic check of inclusion and exclusion criteria.

## Conflicts of Interest

GH received travel grants from Gilead Sciences. NL received speaker fees from Gilead Sciences, Orphalan and Fresenius Kabi; is an advisory board member for Ipsen, received travel grants from Abbvie, Gilead Sciences and Norgine and received research grants from Gilead Sciences and Echosens. MV received a travel grant from Ipsen. GD received speaker fees and travel grants from Roche. BS has been consulting for Boehringer Ingelheim Pharma, Mabwell Therapeutics and Surrozen (prior 24 months). B.S.’s institution UC San Diego has received research support from Axial Biotherapeutics, ChromoLogic, CymaBay Therapeutics, Intercept Pharmaceuticals and Prodigy Biotech (prior 24 months). B.S. is founder of Nterica Bio. AG, SD, SAD, EP, TL, AL and PS declare no conflicts of interest.

## Supporting information


**Table S1:** Cut‐off values used to define steatosis and fibrosis grades according to vibration‐controlled transient elastography. ALD, alcohol‐related liver disease; CAP, controlled‐attenuation parameter; F, fibrosis; MASLD, metabolic dysfunction‐associated steatotic liver disease; S, steatosis; ^a^cut‐off values for LSM using a medium probe; ^b^cut‐off values for LSM using an extra‐large probe (5–8) (see supplementary references).
**Table S2:** Vibration‐controlled transient elastography, biological and non‐invasive predictive scores data. ALD, alcohol‐related liver disease; ALT, alanine aminotransferase; AST, aspartate aminotransferase; CAP, controlled attenuation parameter; Chol, cholesterol; CK, creatin kinase; F, fibrosis stage on transient elastography; FIB‐4, fibrosis‐4 index; FLI, fatty liver index; GGT, gamma‐glutamyltranspeptidase; HDL, high‐density lipoprotein; Hepamet, hepamet fibrosis score; HOMA‐IR, homeostasis model assessment‐estimated insulin resistance; LDL, low‐density lipoprotein; LSM, liver stiffness measurement; MASLD, metabolic dysfunction‐associated steatotic liver disease; NFS, non‐alcoholic fatty liver disease severity score; TCI, total calorie intake; (*) data from non‐diabetic patients exclusively.
**Figure S1:** Diagnostic criteria for steatotic liver disease subtypes and controls. Liver steatosis was diagnosed by abdominal imaging. ALD, alcohol‐related liver disease; BMI, body mass index; F, female; HbA1c, glycated haemoglobin; HDL, high‐density lipoprotein; M, male; MASLD, metabolic dysfunction‐associated steatotic liver disease; MetALD, metabolic and alcohol‐related liver disease; WC, waist circumference (1) (see supplementary references).
**Figure S2:** Main anthropometric and biological data. For anthropometric data, age (A), gender proportions (B) and BMI (C) are represented. For biological data, ALT serum level (D), HOMA‐IR (E) and HDL‐C serum level (F) are represented. ALD, alcohol‐related liver disease; ALT, alanine aminotransferase; BMI, body mass index; HDL‐C, high‐density lipoprotein‐cholesterol; HOMA‐IR, homeostasis model assessment‐estimated insulin resistance; MASLD, metabolic dysfunction‐associated steatotic liver disease. (A) Kruskal–Wallis test; (B) Chi‐square tests; (C–F) One‐way ANOVA; *p‐value < 0.05; **p‐value < 0.01; ****p‐value < 0.0001.
**Figure S3:** Vibration‐controlled transient elastography data. (A) comparison of LSM between groups; (B) proportions of fibrosis grades according to LSM; (C) comparison of CAP between groups; (D) proportions of steatosis grades according to CAP. ALD, alcohol‐related liver disease; CAP, controlled‐attenuation parameter; F, fibrosis grades; LSM, liver stiffness measurement; MASLD, metabolic dysfunction‐associated steatotic liver disease; S, steatosis grades; (A, C) One‐way ANOVA; (B, D) Chi‐square tests; *p‐value < 0.05; ****p‐value < 0.0001.
**Figure S4:** Physical activity habits assessed by the international physical activity questionnaire. (A–C) proportion of calorie expenditure (%) per physical activity types for control (A), MASLD (B) and ALD (C) groups; (D) proportions of calorie expenditure per physical activity types per study groups (Chi‐square tests); (E) comparison of calorie expenditure related to weekly exercise between study groups (One‐way ANOVA); ALD, alcohol‐related liver disease; MASLD, metabolic dysfunction‐associated steatotic liver disease; MET: metabolic equivalent; **p‐value < 0.01; ****p‐value < 0.0001.
**Figure S5:** Dietary habits assessed by the 24‐h recall questionnaire. (A–C) proportions of macronutrients daily intake for control (A), MASLD (B) and ALD (C) groups; (D) proportions of macronutrients daily intake compared between study groups (Chi‐square tests); (E) comparison of total daily caloric intake between study groups (One‐Way ANOVA); **p‐value < 0.01; ****p‐value < 0.0001.
**Figure S6:** Knee extension strength according to laterality in SLD groups. ALD, alcohol‐related liver disease; MASLD, metabolic dysfunction‐associated steatotic liver disease; Nm, Newton‐metre; SLD: steatotic liver disease; paired Student's *t*‐tests, **p‐value < 0.01.
**Figure S7:** Performance and quality of the multiple logistic regression model comparing non‐SLD controls and patients with SLD. Dependent variable was the 25th percentile of the liver frailty index from pooled patients with steatotic liver disease; A: percentages of patients of SLD classified according to the liver frailty index; B: correlation matrix of all variables included in the multivariate analysis model; C: ROC curve; D: multicollinearity screening analysis. AUC: area under the curve; BMI: body mass index; EB: energetic balance; LSM: liver stiffness measurement; SLD: steatotic liver disease; VIF: variation inflation factor.
**Figure S8:** Performance and quality of the multiple logistic regression model comparing MASLD and ALD groups. Dependent variable was the 75th percentile of the liver frailty index from pooled patients with steatotic liver disease; A: percentages of patients with SLD classified according to the liver frailty index; B: correlation matrix of all variables included in the multivariate analysis model; C: ROC curve; D: multicollinearity screening analysis. ALD: alcohol‐related liver disease; AUC: area under the curve; BMI: body mass index; EB: energetic balance; LSM; liver stiffness measurement; MASLD: metabolic dysfunction‐associated steatotic liver disease; SLD: steatotic liver disease; VIF: variation inflation factor.
