# Supplementary material for: Non‐Cirrhotic Steatotic Liver Disease is Associated With Impaired Muscle Function: A Cross‐Sectional Study
Source: JCSM Commun. 2025 Oct 7;8(2):e70012. doi: 10.1002/rco2.70012 (PMC12538381; doi:10.1002/rco2.70012)
Supplement: Supplementary file 1 — Table S1: Cut‐off values used to define steatosis and fibrosis grades according to vibration‐controlled transient elastography. ALD, alcohol‐related liver disease; CAP, controlled‐attenuation parameter; F, fibrosis; MASLD, metabolic dysfunction‐associated steatotic liver disease; S, steatosis; acut‐off values for LSM using a medium probe; bcut‐off values for LSM using an extra‐large probe (5–8) (see supplementary references). Table S2: Vibration‐controlled transient elastography, biological and non‐invasive predictive scores data. ALD, alcohol‐related liver disease; ALT, alanine aminotransferase; AST, aspartate aminotransferase; CAP, controlled attenuation parameter; Chol, cholesterol; CK, creatin kinase; F, fibrosis stage on transient elastography; FIB‐4, fibrosis‐4 index; FLI, fatty liver index; GGT, gamma‐glutamyltranspeptidase; HDL, high‐density lipoprotein; Hepamet, hepamet fibrosis score; HOMA‐IR, homeostasis model assessment‐estimated insulin resistance; LDL, low‐density lipoprotein; LSM, liver stiffness measurement; MASLD, metabolic dysfunction‐associated steatotic liver disease; NFS, non‐alcoholic fatty liver disease severity score; TCI, total calorie intake; (*) data from non‐diabetic patients exclusively. Figure S1: Diagnostic criteria for steatotic liver disease subtypes and controls. Liver steatosis was diagnosed by abdominal imaging. ALD, alcohol‐related liver disease; BMI, body mass index; F, female; HbA1c, glycated haemoglobin; HDL, high‐density lipoprotein; M, male; MASLD, metabolic dysfunction‐associated steatotic liver disease; MetALD, metabolic and alcohol‐related liver disease; WC, waist circumference (1) (see supplementary references). Figure S2: Main anthropometric and biological data. For anthropometric data, age (A), gender proportions (B) and BMI (C) are represented. For biological data, ALT serum level (D), HOMA‐IR (E) and HDL‐C serum level (F) are represented. ALD, alcohol‐related liver disease; ALT, alanine aminotransferase; BMI, body ma [file RCO2-8-e70012-s001.docx]

1. **Supplementary methods**
   1. Diet assessment

Dietary habits were assessed by a dietitian using the 24-hour recall questionnaire (Fig. 1) to record oral calorie intake during the twenty-four hours preceding the questionnaire completion. Food quantity was estimated by showing to the patient previously weighed representative food photographs (1). Food composition in macronutrients expressed in grams and in percentage of total calorie intake was calculated using a dedicated dietetic software (NubelPro^®^). Daily alcohol intake was assessed using the 24-hour recall questionnaire and converted into units per week, one unit of alcohol being equal to ten grams of pure alcohol (2)).

- 1. Physical activity level assessment

Physical activity was assessed using the international physical activity questionnaire (IPAQ) (Fig. 1). Time patients spent exercising in four different active domains (work, travel, domestic, leisure) during the week preceding the questionnaire completion was recorded. Time allocated to perform each exercise in minutes is then multiplied by the metabolic equivalent of a task correlated to its intensity (1 at rest, 3.3 for walking, 4 for moderate and 8 for vigorous exercises). Results are expressed in metabolic equivalent task-minute (MET-min) per week and reflects the energy expenditure (3)).

- 1. Energetic balance assessment

The energetic balance results from the difference between calorie intake and calorie expenditure. Total daily calorie intake was measured using the 24-hour recall questionnaire. Calorie expenditure was calculated by multiplying the basal or resting metabolic rate (RMR) determined with the revised *Harris and Benedict* formula ((4) by the level of physical activity. Physical activity level was quantified by the IPAQ as follows: 1.4 below 600 MET-min per week, 1.6 between 600 and 3000 MET-min per week or 1.8 above 3000 MET-min per week (3)).

1. **Supplementary tables**

|  | **CAP (dB/m)** | | | **LSM (kPa)** | | |
| --- | --- | --- | --- | --- | --- | --- |
| grades | S1 | S2 | S3 | F2 | F3 | F4 |
| MASLD | ≥ 215 | ≥ 252 | ≥ 296 | ≥ 7.8 ^a^  ≥ 6.4 ^b^ | ≥ 12.5 ^a^ ≥ 9.3 ^b^ | ≥ 22.3 ^a^  ≥ 16.0 ^b^ |
| ALD | ≥ 248 | ≥ 268 | ≥ 280 | ≥ 9 | ≥ 12.2 | ≥ 18.5 |

**Table S1. Cut-off values used to define steatosis and fibrosis grades according to vibration-controlled transient elastography.** ALD, alcohol-related liver disease; CAP, controlled-attenuation parameter; F, fibrosis; MASLD, metabolic dysfunction-associated steatotic liver disease; S, steatosis; ^a^ cut-off values for LSM using a medium probe; ^b^ cut-off values for LSM using an extra-large probe ((5–8).

| **variables** | **Controls (N=30)** | | **MASLD (N=75)** | | **ALD (N=75)** | | **p-value** |
| --- | --- | --- | --- | --- | --- | --- | --- |
|  | median | [min;max] | median | [min;max] | median | [min;max] |  |
| **Transient elastography data** | | | | | | | |
| LSM (kPa) | 4.2 | [2.7;6.6] | 8.4 | [2.4;21.2] | 8 | [2.8;17.4] | <0.001^α, β^ |
| F0-F1 – n (%) | 30 (100) | - | 31 (42) | - | 46 (61) | - | <0.001^α, β, γ^ |
| F2 – n (%) | 0 | - | 25 (33) | - | 18 (24) | - | <0.001^α, β^ |
| F3 – n (%) | 0 | - | 19 (25) | - | 11 (15) | - | <0.001^α, β^ |
| CAP (dB/m) | 196 | [124;272] | 328 | [216;400] | 297 | [248;386] | <0.001^α, β, γ^ |
| S0 – n (%) | 24 (80) | - | 0 | - | 0 | - | <0.001^α, β^ |
| S1 – n (%) | 5 (17) | - | 4 (5) | - | 9 (12) | - | 0.160 |
| S2 – n (%) | 1 (3) | - | 15 (20) | - | 11 (15) | - | 0.100 |
| S3 – n (%) | 0 | - | 56 (75) | - | 55 (73) | - | <0.001^α, β^ |
| **Biological data** | | | | | | | |
| Platelets (x10^3^/mm^3^) | 239 | [165;326] | 257 | [73;516] | 214 | [76;425] | 0.001^γ^ |
| Ferritin (µg/l) | 125 | [34;405] | 224 | [15;1474] | 339 | [39;3446] | <0.001^α, β, γ^ |
| Glycaemia (mg/dl) | 91 | [72;111] | 105 | [71;215] | 97 | [61;148] | <0.001^α, β, γ^ |
| Insulin (pmol/l)(*) | 33 | [9.4;125.1] | 112.3 | [25.3;314] | 45.6 | [4;322.7] | <0.001^α, β, γ^ |
| HOMA-IR (*) | 1 | [0.3;4.4] | 3.8 | [0.7;12.9] | 2.2 | [0.1;21.3] | <0.001^α, β, γ^ |
| Albumin (g/l) | 46.5 | [39;54] | 46 | [38;54] | 46 | [41;51] | 0.912 |
| CK (U/l) | 90 | [31;391] | 104.5 | [35;596] | 126 | [36;1540] | 0.043^β^ |
| AST (U/l) | 23 | [12;42] | 30 | [15;84] | 64 | [20;374] | <0.001^α, β, γ^ |
| ALT (U/l) | 16 | [8;56] | 42 | [8;173] | 51 | [10;210] | <0.001^α, β, γ^ |
| GGT (U/l) | 14 | [7;40] | 40 | [10;268] | 177 | [14;3131] | <.001^α, β, γ^ |
| Alkaline Phosphatase (U/l) | 57.5 | [21;118] | 71.5 | [28;202] | 87.5 | [40;227] | <0.001^β, γ^ |
| Total chol (mg/dl) | 187 | [143;255] | 178 | [67;267] | 222 | [151;377] | <0.001^β, γ^ |
| LDL-Chol (mg/dl) | 107 | [63;155] | 99 | [24;191] | 115 | [36;242] | 0.014 |
| HDL-Chol (mg/dl) | 69 | [48;150] | 47 | [17;75] | 72 | [33;171] | <0.001^α, γ^ |
| Triglycerides (mg/dl) | 61 | [38;94] | 142 | [53;386] | 120 | [45;766] | <0.001^α, β^ |
| **Bio-clinical non-invasive scores** | | | | | | | |
| Agile3+ | 0.11 | [0.02;0.38] | 0.27 | [0.03;0.96] | 0.35 | [0.01;0.91] | <0.001^α, β^ |
| FLI | 8 | [1;39] | 86 | [10;100] | 80 | [4;100] | <0.001^α, β^ |
| FIB-4 | 1.2 | [0.4;1.8] | 1.05 | [0.3;4.7] | 1.8 | [0.3;11.6] | <0.001^β, γ^ |
| NFS | -2.4 | [-4.6;-0.2] | -1.7 | [-4.8;-0.6] | -1.9 | [-4.8;0.6] | 0.010^α^ |
| Hepamet | 0.01 | [0;0.1] | 0.06 | [0;1] | 0.04 | [0;0.5] | <0.001^α, β^ |
| **energetic balance** | | | | | | | |
| basal metabolism (kcal) | 1431 | [1182 ; 1879] | 1752 | [1287 ; 2487] | 1729 | [1152 ; 2164] | <0.001^α, β^ |
| caloric need (kcal) | 2491 | [2004 ; 3382] | 2869 | [1802 ; 4126] | 2713 | [1719 ; 3639] | 0.103 |
| energetic balance (% TCI) | -21.6 | [-72.5 ; 22.9] | -38.8 | [-86.1 ; 15.1] | -8.9 | [-79.8 ; 104] | <0.001^γ^ |

**Table S2.** **Vibration-controlled transient elastography, biological and non-invasive predictive scores data**. ALD, alcohol-related liver disease; ALT, alanine aminotransferase; AST, aspartate aminotransferase; CAP, controlled attenuation parameter; Chol, cholesterol; CK, creatin kinase; F, fibrosis stage on transient elastography; FIB-4, fibrosis-4 index; FLI, fatty liver index; GGT, gamma-glutamyltranspeptidase; HDL, high-density lipoprotein; Hepamet, hepamet fibrosis score; HOMA-IR, homeostasis model assessment-estimated insulin resistance; LDL, low-density lipoprotein; LSM, liver stiffness measurement; MASLD, metabolic dysfunction-associated steatotic liver disease; NFS, non-alcoholic fatty liver disease severity score; TCI, total calorie intake; (*) data from non-diabetic patients exclusively.

1. **Supplementary figures**

**
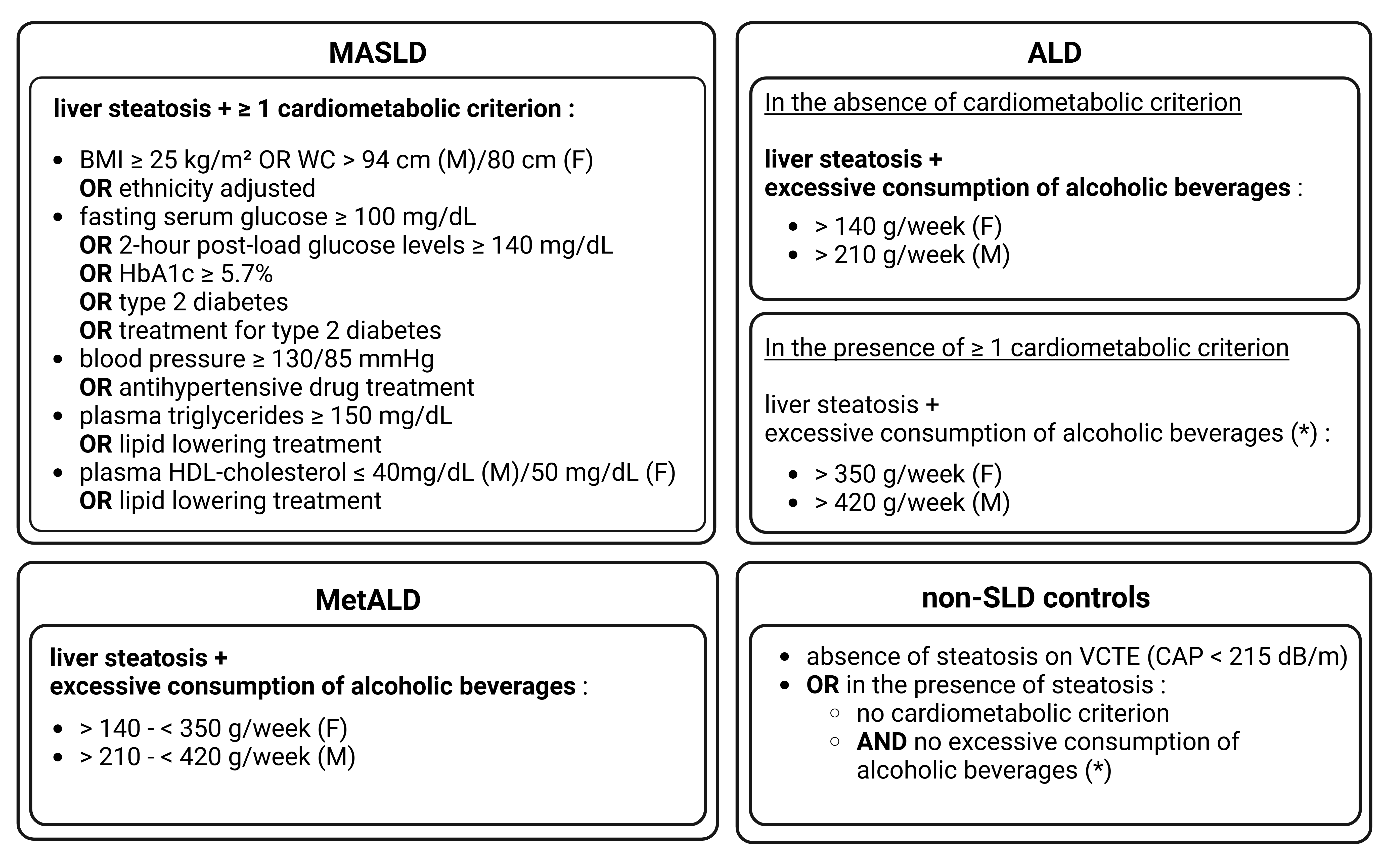
**

**Fig. S1. Diagnostic criteria for steatotic liver disease subtypes and controls.** Liver steatosis was diagnosed by abdominal imaging. ALD, alcohol-related liver disease; BMI, body mass index; F, female; HbA1c, glycated haemoglobin; HDL, high-density lipoprotein; M, male; MASLD, metabolic dysfunction-associated steatotic liver disease; MetALD, metabolic and alcohol-related liver disease; WC, waist circumference (1).

**
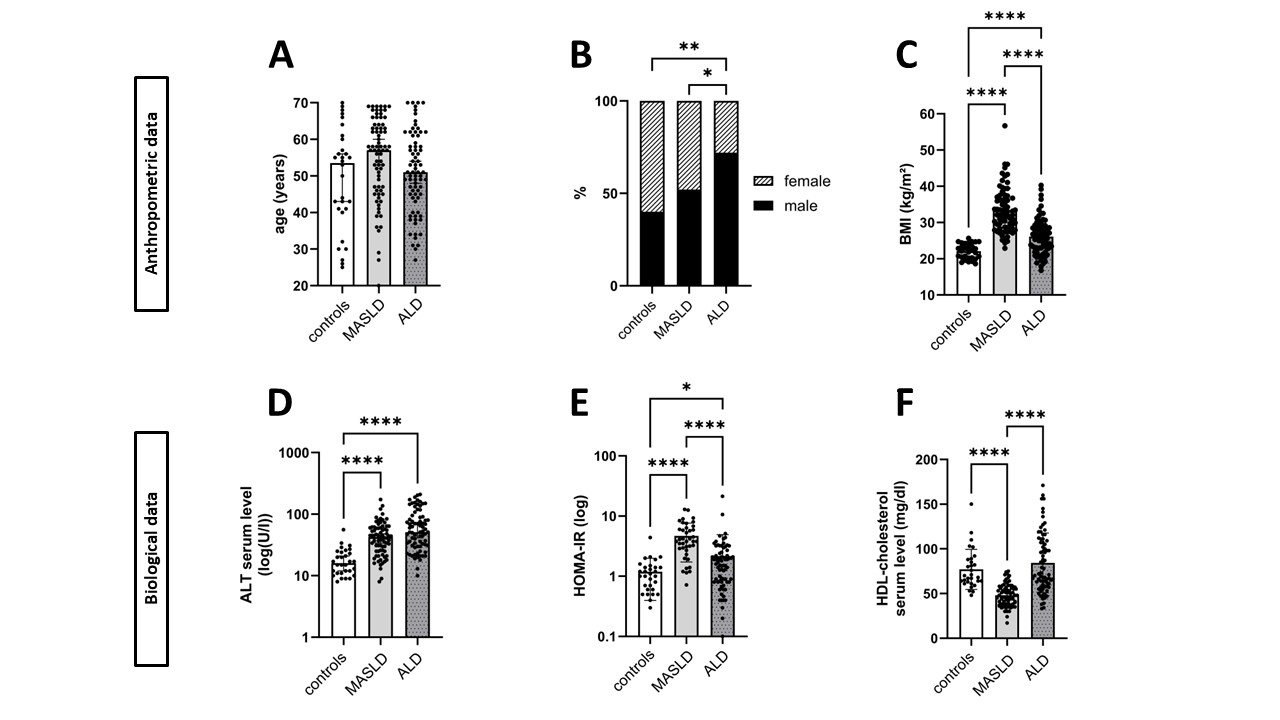
**

**Fig. S2. Main anthropometric and biological data.** For anthropometric data, age (A), gender proportions (B) and BMI (C) are represented. For biological data, ALT serum level (D), HOMA-IR (E) and HDL-C serum level (F) are represented. ALD, alcohol-related liver disease; ALT, alanine aminotransferase; BMI, body mass index; HDL-C, high-density lipoprotein-cholesterol; HOMA-IR, homeostasis model assessment-estimated insulin resistance; MASLD, metabolic dysfunction-associated steatotic liver disease. (A) Kruskal-Wallis test; (B) Chi-square tests; (C-F) One-way ANOVA; * p-value < 0.05; ** p-value < 0.01; **** p-value < 0.0001.

**
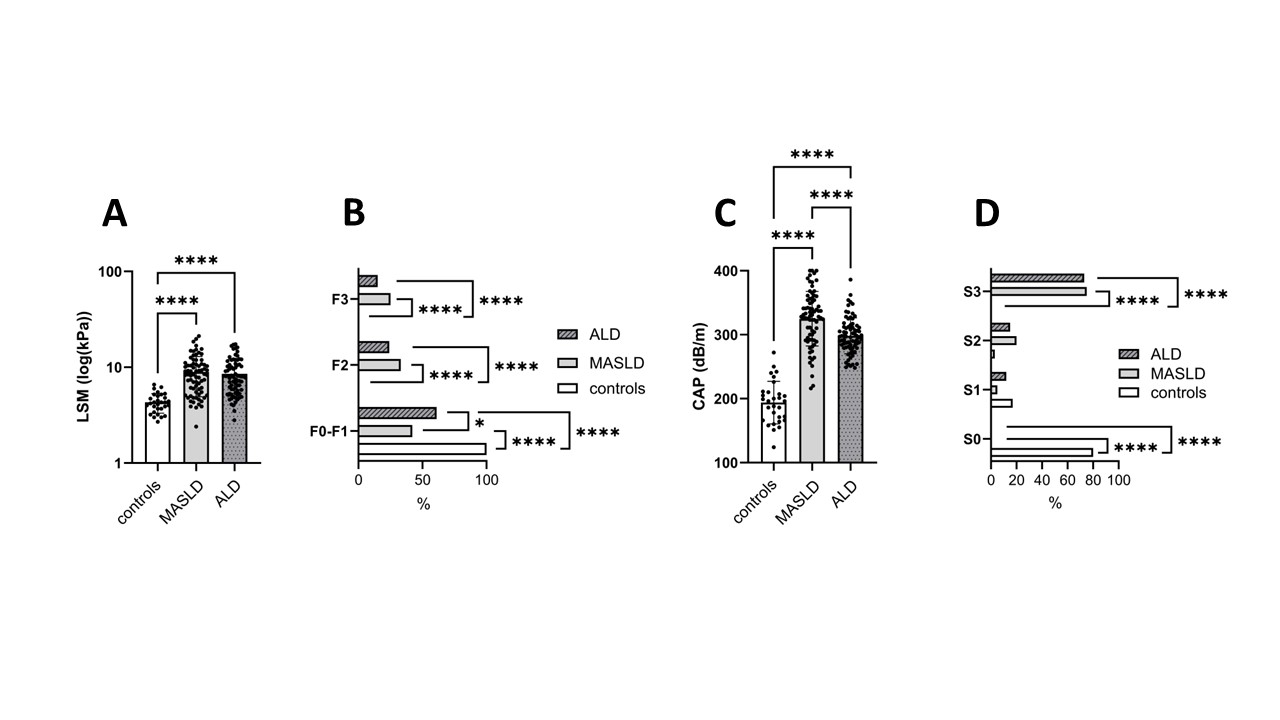
**

**Fig. S3. Vibration-controlled transient elastography data.** (A) comparison of LSM between groups; (B) proportions of fibrosis grades according to LSM; (C) comparison of CAP between groups; (D) proportions of steatosis grades according to CAP. ALD, alcohol-related liver disease; CAP, controlled-attenuation parameter; F, fibrosis grades; LSM, liver stiffness measurement; MASLD, metabolic dysfunction-associated steatotic liver disease; S, steatosis grades; (A, C) One-way ANOVA; (B, D) Chi-square tests; * p-value < 0.05; **** p-value < 0.0001.


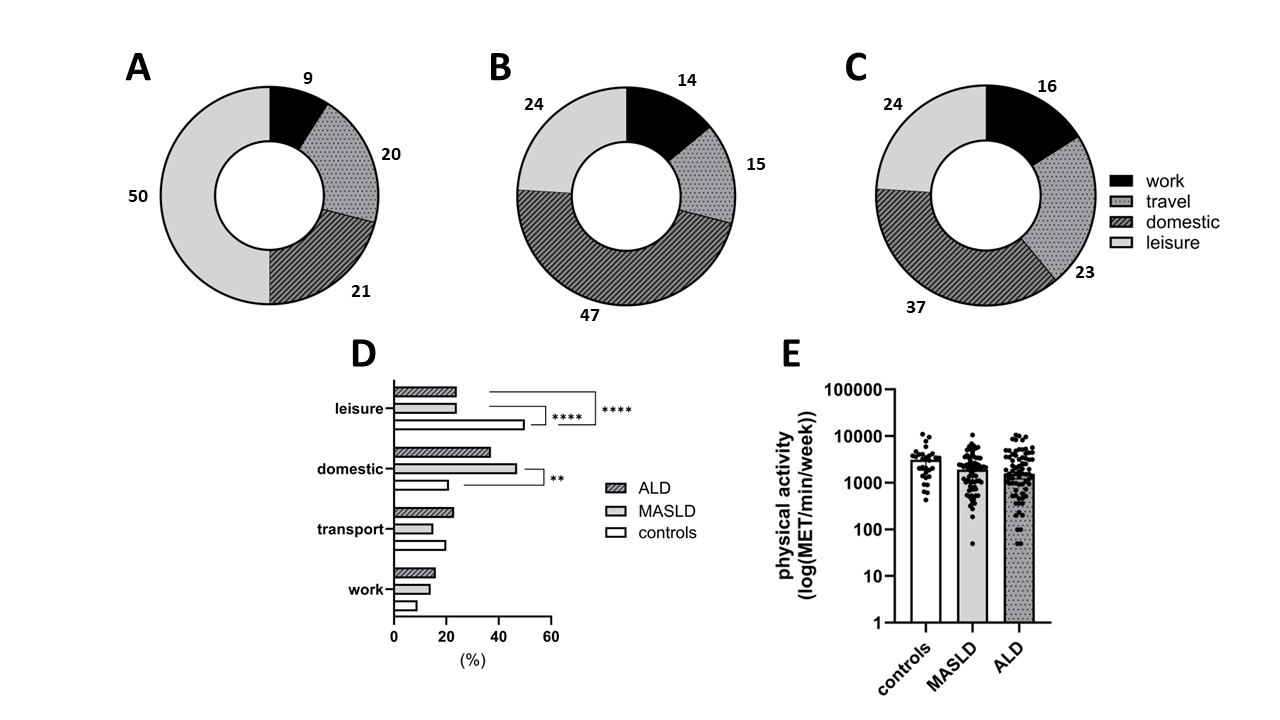


**Fig. S4. Physical activity habits assessed by the international physical activity questionnaire.** (A-C) proportion of calorie expenditure (%) per physical activity types for control (A), MASLD (B) and ALD (C) groups; (D) proportions of calorie expenditure per physical activity types per study groups (Chi-square tests); (E) comparison of calorie expenditure related to weekly exercise between study groups (One-way ANOVA); ALD, alcohol-related liver disease; MASLD, metabolic dysfunction-associated steatotic liver disease; MET: metabolic equivalent; ** p-value < 0.01; **** p-value < 0.0001.


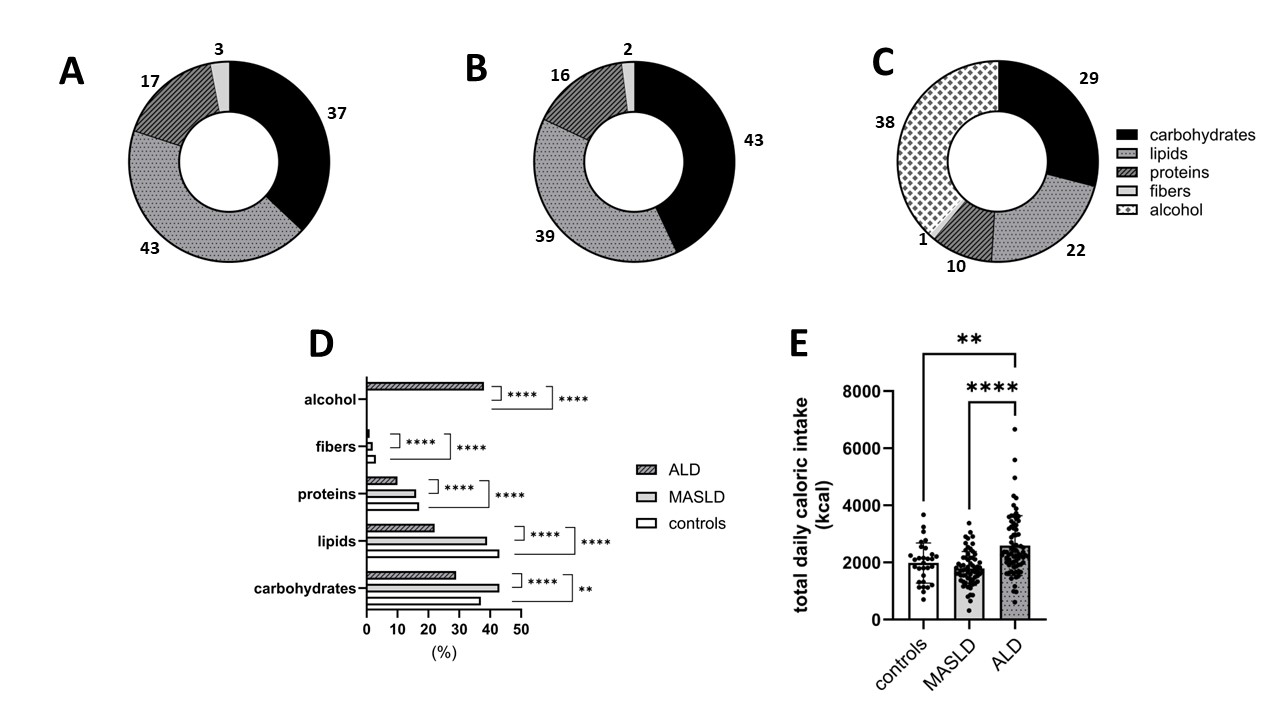


**Fig. S5. Dietary habits assessed by the 24-hour recall questionnaire.** (A-C) proportions of macronutrients daily intake for control (A), MASLD (B) and ALD (C) groups; (D) proportions of macronutrients daily intake compared between study groups (Chi-square tests); (E) comparison of total daily caloric intake between study groups (One-Way ANOVA); ** p-value < 0.01; **** p-value < 0.0001.


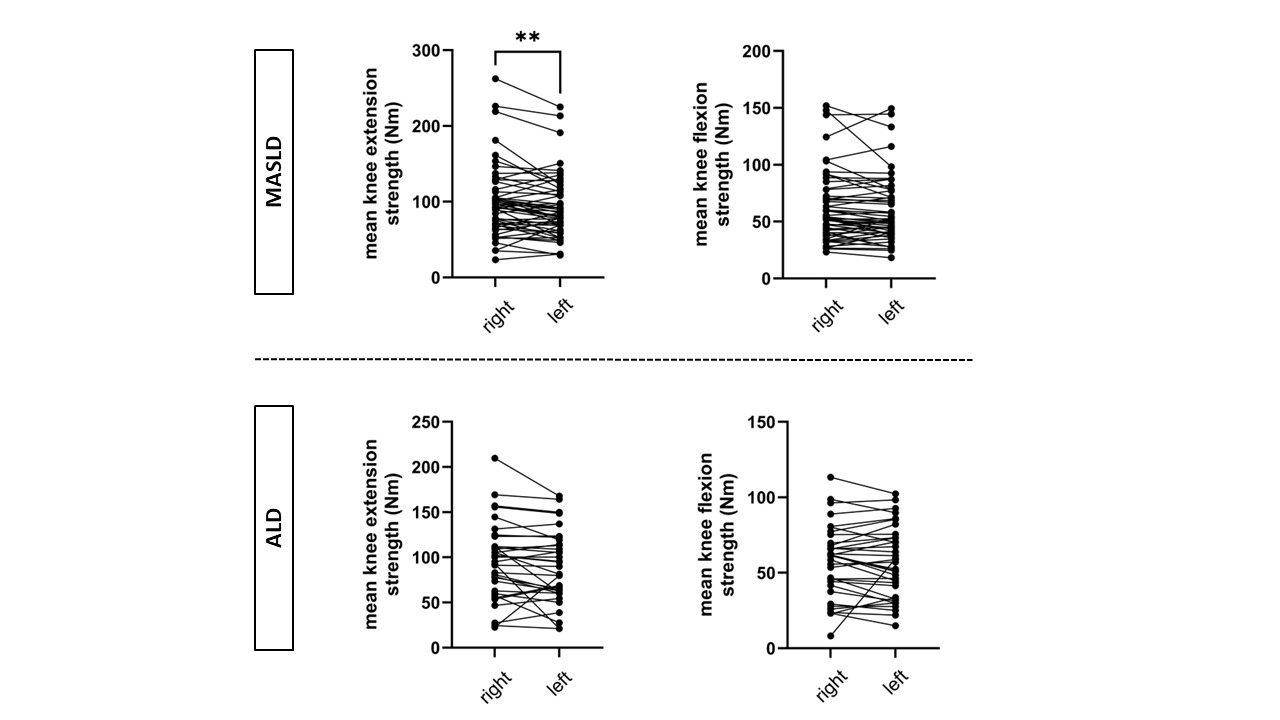


**Fig. S6. Knee extension strength according to laterality in SLD groups.** ALD, alcohol-related liver disease; MASLD, metabolic dysfunction-associated steatotic liver disease; Nm, Newton-meter; SLD: steatotic liver disease; Paired Student’s t-tests, ** p-value < 0.01.


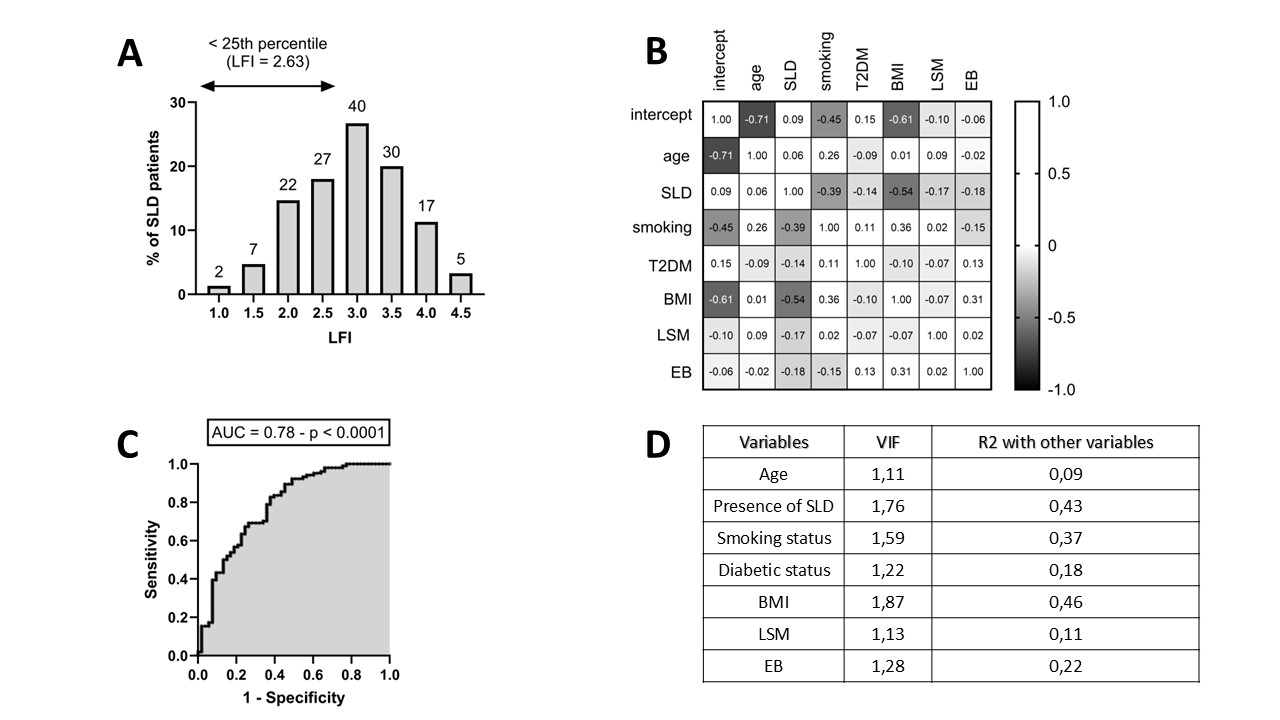


**Fig. S7. Performance and quality of the multiple logistic regression model comparing non-SLD controls and patients with SLD.** Dependent variable was the 25^th^ percentile of the liver frailty index from pooled patients with steatotic liver disease; A: percentages of patients of SLD classified according to the liver frailty index; B: correlation matrix of all variables included in the multivariate analysis model; C: ROC curve; D: multicollinearity screening analysis. AUC: area under the curve; BMI: body mass index; EB: energetic balance; LSM: liver stiffness measurement; SLD: steatotic liver disease; VIF: variation inflation factor.


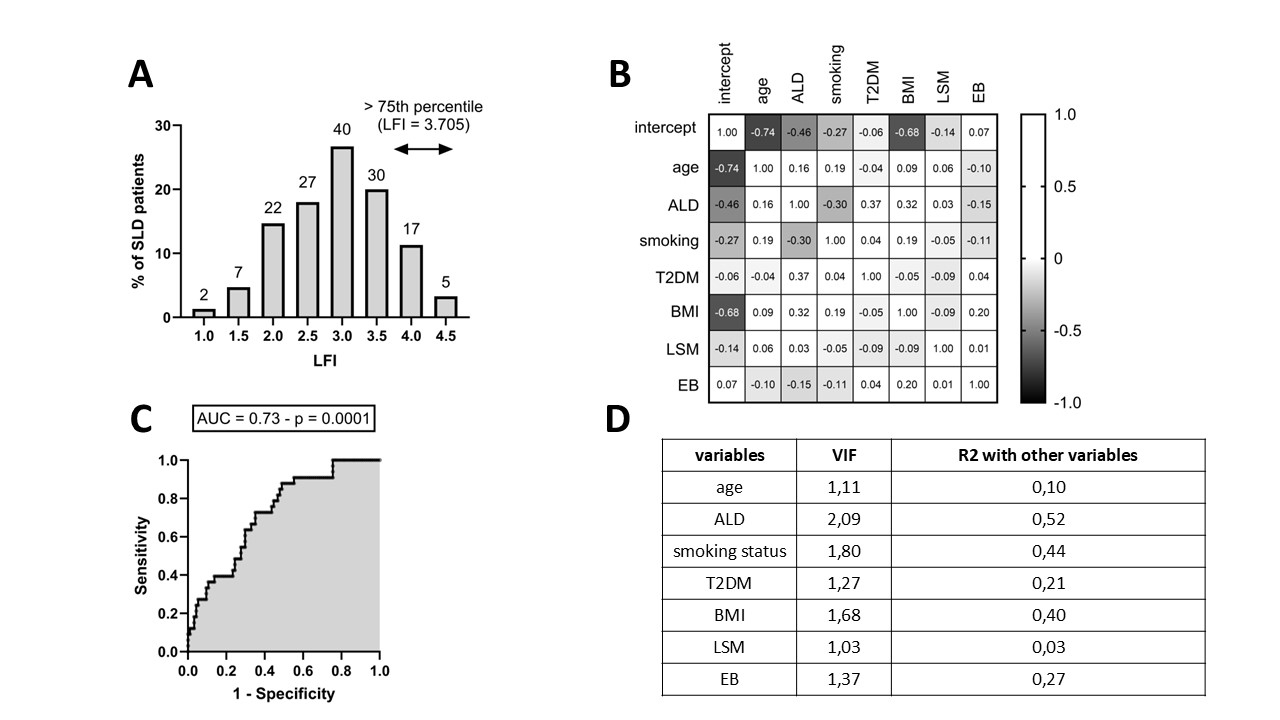


**Fig. S8. Performance and quality of the multiple logistic regression model comparing MASLD and ALD groups.** Dependent variable was the 75^th^ percentile of the liver frailty nidex from pooled patients with steatotic liver disease; A: percentages of patients with SLD classified according to the liver frailty index; B: correlation matrix of all variables included in the multivariate analysis model; C: ROC curve; D: multicollinearity screening analysis. ALD: alcohol-related liver disease; AUC: area under the curve; BMI: body mass index; EB: energetic balance; LSM; liver stiffness measurement; MASLD: metabolic dysfunction-associated steatotic iver disease; SLD: steatotic liver disease; VIF: variation inflation factor.

1. **Supplementary references**

1. Hercberg S, Galan P, Preziosi P, Bertrais S, Mennen L, Malvy D, et al. The SU.VI.MAX Study. Arch Intern Med. 2004 Nov 22;164(21):2335.

2. Gordon H. Detection of alcoholic liver disease. World J Gastroenterol. 2001;7(3):297.

3. Craig CL, Marshall AL, Sjöström M, Bauman AE, Booth ML, Ainsworth BE, et al. International Physical Activity Questionnaire: 12-Country Reliability and Validity. Med Sci Sports Exerc. 2003 Aug;35(8):1381–95.

4. Pavlidou E, Papadopoulou SK, Seroglou K, Giaginis C. Revised Harris–Benedict Equation: New Human Resting Metabolic Rate Equation. Metabolites. 2023 Jan 28;13(2):189.

5. de Lédinghen V, Vergniol J, Foucher J, Merrouche W, le Bail B. Non‐invasive diagnosis of liver steatosis using controlled attenuation parameter ( CAP ) and transient elastography. Liver International. 2012 Jul 6;32(6):911–8.

6. Myers RP, Pomier-Layrargues G, Kirsch R, Pollett A, Duarte-Rojo A, Wong D, et al. Feasibility and diagnostic performance of the FibroScan XL probe for liver stiffness measurement in overweight and obese patients. Hepatology. 2012 Jan;55(1):199–208.

7. Myers RP, Pomier-Layrargues G, Kirsch R, Pollett A, Beaton M, Levstik M, et al. Discordance in fibrosis staging between liver biopsy and transient elastography using the FibroScan XL probe. J Hepatol. 2012 Mar;56(3):564–70.

8. Nguyen-Khac E, Thiele M, Voican C, Nahon P, Moreno C, Boursier J, et al. Non-invasive diagnosis of liver fibrosis in patients with alcohol-related liver disease by transient elastography: an individual patient data meta-analysis. Lancet Gastroenterol Hepatol. 2018 Sep;3(9):614–25.

1. **STROBE checklist (version 4)**

|  | Item No | Recommendation |
| --- | --- | --- |
| **Title and abstract** | 1 | (*a*) Indicate the study’s design with a commonly used term in the title or the abstract |
|  |  | (*b*) Provide in the abstract an informative and balanced summary of what was done and what was found |
| Introduction | | |
| Background/rationale | 2 | Explain the scientific background and rationale for the investigation being reported |
| Objectives | 3 | State specific objectives, including any prespecified hypotheses |
| Methods | | |
| Study design | 4 | Present key elements of study design early in the paper |
| Setting | 5 | Describe the setting, locations, and relevant dates, including periods of recruitment, exposure, follow-up, and data collection |
| Participants | 6 | (*a*) *Cohort study*—Give the eligibility criteria, and the sources and methods of selection of participants. Describe methods of follow-up  *Case-control study*—Give the eligibility criteria, and the sources and methods of case ascertainment and control selection. Give the rationale for the choice of cases and controls  *Cross-sectional study*—Give the eligibility criteria, and the sources and methods of selection of participants |
|  |  | (*b*) *Cohort study*—For matched studies, give matching criteria and number of exposed and unexposed  *Case-control study*—For matched studies, give matching criteria and the number of controls per case |
| Variables | 7 | Clearly define all outcomes, exposures, predictors, potential confounders, and effect modifiers. Give diagnostic criteria, if applicable |
| Data sources/ measurement | 8* | For each variable of interest, give sources of data and details of methods of assessment (measurement). Describe comparability of assessment methods if there is more than one group |
| Bias | 9 | Describe any efforts to address potential sources of bias |
| Study size | 10 | Explain how the study size was arrived at |
| Quantitative variables | 11 | Explain how quantitative variables were handled in the analyses. If applicable, describe which groupings were chosen and why |
| Statistical methods | 12 | (*a*) Describe all statistical methods, including those used to control for confounding |
|  |  | (*b*) Describe any methods used to examine subgroups and interactions |
|  |  | (*c*) Explain how missing data were addressed |
|  |  | (*d*) *Cohort study*—If applicable, explain how loss to follow-up was addressed  *Case-control study*—If applicable, explain how matching of cases and controls was addressed  *Cross-sectional study*—If applicable, describe analytical methods taking account of sampling strategy |
|  |  | (*e*) Describe any sensitivity analyses |

1. Continued on next page

| Results | | |
| --- | --- | --- |
| Participants | 13* | (a) Report numbers of individuals at each stage of study—eg numbers potentially eligible, examined for eligibility, confirmed eligible, included in the study, completing follow-up, and analysed |
|  |  | (b) Give reasons for non-participation at each stage |
|  |  | (c) Consider use of a flow diagram |
| Descriptive data | 14* | (a) Give characteristics of study participants (eg demographic, clinical, social) and information on exposures and potential confounders |
|  |  | (b) Indicate number of participants with missing data for each variable of interest |
|  |  | (c) *Cohort study*—Summarise follow-up time (eg, average and total amount) |
| Outcome data | 15* | *Cohort study*—Report numbers of outcome events or summary measures over time |
|  |  | *Case-control study—*Report numbers in each exposure category, or summary measures of exposure |
|  |  | *Cross-sectional study—*Report numbers of outcome events or summary measures |
| Main results | 16 | (*a*) Give unadjusted estimates and, if applicable, confounder-adjusted estimates and their precision (eg, 95% confidence interval). Make clear which confounders were adjusted for and why they were included |
|  |  | (*b*) Report category boundaries when continuous variables were categorized |
|  |  | (*c*) If relevant, consider translating estimates of relative risk into absolute risk for a meaningful time period |
| Other analyses | 17 | Report other analyses done—eg analyses of subgroups and interactions, and sensitivity analyses |
| Discussion | | |
| Key results | 18 | Summarise key results with reference to study objectives |
| Limitations | 19 | Discuss limitations of the study, taking into account sources of potential bias or imprecision. Discuss both direction and magnitude of any potential bias |
| Interpretation | 20 | Give a cautious overall interpretation of results considering objectives, limitations, multiplicity of analyses, results from similar studies, and other relevant evidence |
| Generalisability | 21 | Discuss the generalisability (external validity) of the study results |
| Other information | | |
| Funding | 22 | Give the source of funding and the role of the funders for the present study and, if applicable, for the original study on which the present article is based |

1. *Give information separately for cases and controls in case-control studies and, if applicable, for exposed and unexposed groups in cohort and cross-sectional studies.
2. **Note:** An Explanation and Elaboration article discusses each checklist item and gives methodological background and published examples of transparent reporting. The STROBE checklist is best used in conjunction with this article (freely available on the Web sites of PLoS Medicine at http://www.plosmedicine.org/, Annals of Internal Medicine at http://www.annals.org/, and Epidemiology at http://www.epidem.com/). Information on the STROBE Initiative is available at www.strobe-statement.org.
